# Annotations

**Published:** 1892-10-01

**Authors:** 


### ANNOTATIONS.

**Shall Doctors take the Lead?**

By way of illustration and practical confirmation of Mr. Gladstone's expressed conviction that doctors hold an advanced place in our advancing civilization, and are probably destined for still more prominent leadership, the following quotation from a recent article in *The Leisure Hour* on the "Statesmen of Europe" is well worthy of consideration. Says the writer of the article: "It is doubtless the international character of science that has helped to give Virchow his cosmopolitan comprehension of questions outside the range of his own speciality, and has enabled him to bring this note into his political labours, in which the parochial tone is far too apt to prevail. And this cosmopolitan note is of the greatest value in Germany, where particularism . . . has always had a tendency to predominate, leading to those race hatreds which have found such an ugly expression in the anti-Semitic movement, of which Virchow has been a bitter foe. For all this, however, none of Virchow's opponents have dared to call him anti-patriotic, liberal though they be in bestowing that term. They are forced to recognise that he is only opposed to noxious or absurd exaggerations of patriotic feeling, such as are too much the fashion in the Germany of to-day. . . . He does not speak often in the Reichstag, but when he does it is with weight, objectivity, clearness, and judgment, and his hearers feel that the words uttered are the result of real and calm reflection." This is an admirable description of an admirable temper of mind. Virchow, as all medical men know, is not only a medical scientist, but a loyal medical man. He carries the methods of the laboratory and of the clinique into the political arena. What other methods could be better, or so good? For prejudice and passion he substitutes reason; for the wilfulness of those who rule he substitutes the well-being of those who are ruled. This surely should be the elementary philosophy of all government. But when has it ever been so? It will be so when the methods of the laboratory and of the clinique are adopted in legislative assemblies. Mr. Gladstone spoke prophetically when he said that doctors had a great part to play in the leadership of the future. There is, of course, always the possibility that medical men, if tried with leadership, might fall away from their own principles and methods. But it will be well to wait until they do this before giving them credit for such unscientific unworthiness.

**Singing-Masters Science.**

Slowly—very slowly—but surely, a sense of humour is being developed in the medical profession. We are permitted nowadays to indulge in a little mild laughter at professors and learned persons if, by some rare chance, they make themselves ridiculous. Our transatlantic brethren of the great Western continent are in this, as in so many other respects, a long way ahead of us. They have gone so far as the production of "Imaginary Conversations," in which members of one of the professions—not, of course, the medical—are made to cut very amusing figures in medical eyes. Here is one such conversation, which is vouched for by the *British Medical Journal*. The occasion is the return of a lady with her youthful daughter from a visit to one of several professors of singing, each of whom had been requested to test the little girl's voice. The professor is nothing if not scientific, and the science of half-a-dozen singing-masters had bewildered the poor lady to such a degree that she had finally decided that her daughter should give up the idea of singing altogether; and thus the too scientific professors were all deprived of a profitable client. "The first professor," repeated the lady to her husband, "said that Almira sings too much with her borax. If she keeps on she will get digestion of the lungs. He said she ought to try the abominable breathing, and practise solfudgery. Then the next teacher told me she ought to sing with her diagram, and not smother her voice in the sarcophagus. Then the next he poked a looking-glass down her throat, and said the phalanx was too small, and the typhoid bone and the polyglottis were in a bad way; and I never knew that Almira had so many things down her throat, and I am afraid to let her sing any more for fear it will kill the poor girl." This is delightfully foolish. Of course the good lady made a sad hash of her scientific vocabulary. But why does a singing-master want to be talking about the thorax, and abdominal breathing, and the diaphragm, and the pharynx, and the œsophagus, and the hyoid bone and the epiglottis? It can only be a spirit of the most vulgar display which prompts the use of such meaningless composities. To the credit of medical men it must be said that they never overwhelm their patients with sounding medical or anatomical terms. To the doctor the thorax is the chest, the pharynx the throat, and so on. It may almost invariably be taken for granted that the man who makes a great display of learning, especially to the unlearned, is as destitute of real learning as he is of good sense and good taste.

**The Doctor, the Workshop, and the State.**

There are two sorts of doctors—those who love patients for the sake of their fees, and those who cherish the grand ambition to bring in the millenium of perfect health for every human creature. Similarly, there are two sorts of statesmen—those who seek office for the sake of salary and position, and those who aim at making every man, woman, and child prosperous, healthy, and happy. The modern statesman is not unlike the conductor of a great business enterprise, a kind of political Whiteley. He must offer something which the public requires and will accept, or else he must, metaphorically, put up his shutters. There is a great deal of new ground to break for the statesmanship of this country. The scientific method is opening out new worlds. Statesmen are shy of the scientific method. They might just as well be shy of a fire in winter, or of the table at dinner-time. They have either to take up the methods of science and apply them to the work of the state, or to stand aside as antiquated, incompetent, and blind noodles. The methods of science are as much in the air as the new fashions in ladies' bonnets. They must be adopted. Dr. Arlidgel has broken new ground on his own account in a book which he calls "The Hygiene: Diseases and Mortality of Occupations." He tells us how various classes of workers live, and how they die. According to him, the workshop and the factory are more dangerous to life than the sea, and sometimes even than the battlefield. Many workers—printers, potters, factory men, women and children, blacksmiths, puddlers, masons, and a whole class, counting its numbers by millions, have health destroyed and life shortened by the mere stress and poison of their daily toil. Some of these workers do not live out half their days. Dr. Arlidge is no Government official publishing an annual report, unconscious of the terrible nature of his own facts. He understands what he is doing. He is doing his present work because he understands it. He has gone down into the places he speaks of, and he has come up again to tell the world, from the scientific, not the philanthropic standpoint, what he has seen, and what it all means. It means that, as a nation, we are terribly stupid and dull. We allow a few people to exploit as for their profit and ambition. But we must put a stop to such madness. Science, in the shape of Dr. Arlidge and other like-minded persons, is ready to help us. We must seek their help through our statesmen, and we must make it possible for our working men to live out their full time, and to have a reasonable degree of enjoyment in life.

